# Pathways to Diagnose Infectious Pulmonary Vascular Disease in Rural Mozambique

**DOI:** 10.3390/idr17050116

**Published:** 2025-09-15

**Authors:** Yolanda Sabino, Cizália Ribeiro, Joshua Mungue, Ana Olga Mocumbi

**Affiliations:** 1 Hospital Geral da Polana Caniço, Clínica da Universidade, Maputo 3000, Mozambique; y.marcelino@ymail.com; 2 Hospital Rural de Nhamatanda, Vila de Nhamatanda, Sofala 2106, Mozambique; cizaliaribeiro123@gmail.com (C.R.); jochuamungue@gmail.com (J.M.); 3 Instituto Nacional de Saúde, Estrada Nacional, Nr. 1, Marracuene 1008, Mozambique; 4 Faculdade de Medicina, Universidade Eduardo Mondlane, Maputo 1102, Mozambique

**Keywords:** infectious pulmonary vascular disease, diagnosis, schistosomiasis, Africa

## Abstract

**Background:** Schistosomiasis, HIV, and tuberculosis frequently lead to pulmonary hypertension in low- and middle-income countries. Lack of specific testing and limited access to right heart catheterization hamper confirmation of the etiology of pulmonary hypertension due to schistosomiasis. In addition, low health literacy and poor socioeconomic status further compromise prevention, early diagnosis, and treatment. Clinical algorithms for early screening, including hand-held echocardiography and point-of-care testing performed by non-specialists, are needed in rural Sub-Saharan Africa to decentralize care and improve outcomes. **Methods:** We describe a case of pulmonary hypertension diagnosed in a child living in Mozambique, to discuss the challenges for the diagnosis of infectious pulmonary arterial hypertension in rural settings in Africa, based on a short literature review.

## 1. Introduction

In low- and middle-income countries (LMIC), HIV, tuberculosis, and schistosomiasis may lead to pulmonary hypertension (PH) [1]. Extensive workup for the cause of PH is mandatory to accurately classify all types [2] and identify factors involved, a key step to reducing PH burden in LMIC. On the other hand, tracking of diagnostic practices is key to identify areas of improvement for early diagnosis of infectious pulmonary vascular disease (iPVD). Early diagnosis facilitates the initiation of treatment minimizing symptom burden, optimizes the patient’s biochemical, hemodynamic, and functional profile, and curtails adverse events [3]. In LMLC, like Mozambique having diagnostic limitations, pulmonary arterial hypertension (PAH) requires advanced tools like echocardiography, right heart catheterization, and pulmonary function tests—rarely available outside tertiary centers; schistosomiasis diagnosis often relies on microscopy of urine or stool, which has low sensitivity, especially in chronic or low-intensity infections; newer diagnostics (e.g., antigen detection, PCR) are promising but not widely deployed in rural Mozambique. Children with fatigue, dyspnea, and stunted growth may raise suspicion for PH or chronic schistosomiasis—but these symptoms are nonspecific and easily attributed to other conditions. Without confirmatory tests, clinicians must rely on clinical judgment, which can lead to underdiagnosis or misclassification. Rural children are frequently exposed to infested water sources, making schistosomiasis a plausible etiology even without lab confirmation. Chronic schistosomiasis can lead to vascular remodeling and PH, but this link is rarely tracked due to fragmented care pathways. Without early recognition, children may present late with irreversible complications—a missed opportunity for prevention [4]. In Mozambique and similar LMICs, the landscape shifts dramatically: Unoperated Congenital Heart Disease—due to delayed diagnosis and limited surgical capacity [5]; Advanced Rheumatic Heart Disease (RHD)—leading cause of PH in children, often presenting late with severe valvular damage; Schistosomiasis-Associated PH—particularly hepatosplenic schistosomiasis (*S. mansoni*), leading to portosystemic shunting and egg embolization in pulmonary vasculature [6]; Sickle Cell Disease—contributes to chronic hemolysis and endothelial dysfunction [7]; Persistent Pulmonary Hypertension of the Newborn (PPHN)—often linked to prematurity and perinatal complications, with limited neonatal intensive care support. These causes reflect a multifactorial burden, often compounded by late presentation, underdiagnosis, and limited access to specialized care [8]. Health system challenges in LMICs have structural and systemic barriers like delayed diagnosis (lack of routine echocardiography and catheterization services), workforce shortages (few pediatric cardiologists, pulmonologists, and intensivists), limited access to medications (pulmonary vasodilators (e.g., sildenafil, bosentan) are often unavailable or unaffordable), fragmented referral pathways (weak integration between primary care and tertiary centers), and inadequate data systems (sparse registries and surveillance for pediatric PH [9]. The pathophysiology of pulmonary hypertension (PH) caused by schistosomiasis is an intersection of parasitology, immunology, and vascular biology. It is a cascade of immune-driven remodeling that transforms the pulmonary vasculature which starts with egg migration and vascular seeding—*Schistosoma* eggs reach the lungs via portocaval shunts in patients with hepatosplenic disease. These eggs lodge in pre-capillary pulmonary vessels, triggering local inflammation. The immune system mounts a Th2-dominant response: IL-4, IL-5, and IL-13 are upregulated; eosinophils, macrophages, and fibrocytes infiltrate the lung tissue. This leads to granuloma formation around the eggs, similar to hepatic pathology. After this, vascular remodeling occurs with chronic inflammation activating TGF-β signaling, especially via Thrombospondin-1 (TSP-1). TGF-β promotes smooth muscle proliferation, intimal fibrosis, and media thickening. These changes narrow the pulmonary arteries, increasing resistance and pressure. Hemodynamic consequences such as sustained vascular remodeling lead to elevated pulmonary artery pressure, right ventricular hypertrophy, and, eventually, right heart failure (cor pulmonale) [6].

## 2. Case Description of Pathway to Diagnosis

**Figure 1:**
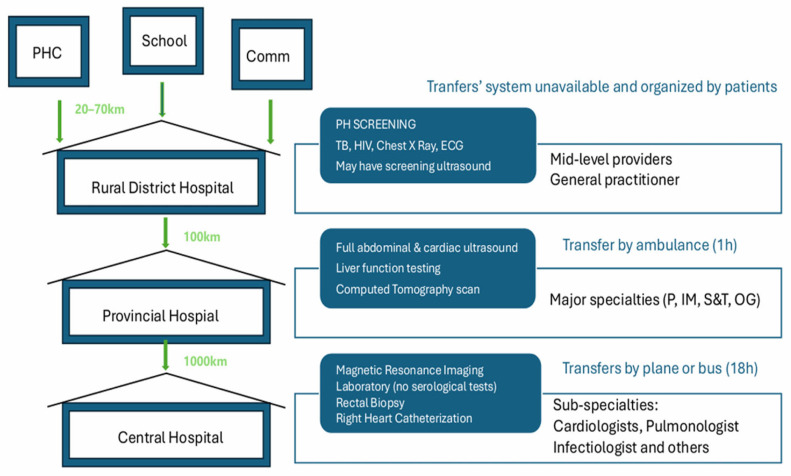
Pathways to diagnosis, iPVD. Central Illustration: The four levels of the health system in Mozambique are represented, showing the relevant resources available at each of them. Referral follows a standardized path within the national health service (green arrows) but may be delayed by needs such as obtaining the national identification card and securing a family member to travel with the child may take several months, and are usually determined by parents needing to stop working in order to travel with the children. PHC—health center; Comm—community; TB—tuberculosis; ECG—electrocardiography; P—pediatrics; IM = internal medicine; S&T—surgery and traumatology; OG—obstetrics and gynecology.

A 10-year-old boy from a family of low socio-economic status living in a remote rural area in central Mozambique was at the district hospital in February/2025, brought by a primary school teacher previously trained in screening of cardiovascular diseases in children nine months before. While visiting a remote area of the district, the teacher suspected cardiac disease in that boy and scheduled an appointment with a cardiologist, with quarterly visits to the rural clinic to support the local non-specialist team. Despite having all routine vaccines, failure to thrive, and repeated pulmonary infections since four months of age, the child had not been seen by a doctor. No clear antecedent of schistosomiasis or TB was found. The boy had no cyanosis (sat O_2_ 95%) or dyspnea at rest and had a quiet precordium, a mildly audible tricuspid murmur, and mild hepatosplenomegaly. Moderate exercise provoked extreme tiredness, chest pain, 87% cyanosis, and tachycardia. The chest X-ray did not reveal signs of pulmonary tuberculosis; the TB GenExpert and HIV test were negative. An ECG revealed sinus tachycardia, right axis deviation, P pulmonale, right bundle branch block, and right ventricular hypertrophy. On transthoracic echocardiography, features of PH were present: dilatation and hypertrophy of the right ventricle, severe tricuspid regurgitation, and pulmonary artery dilatation, with no evidence of congenital heart disease, structural valve disease, or left heart disease. Because further procedures for diagnosis of pulmonary vascular disease are unavailable in this rural health facility, the patient needs to be transferred either to a referral hospital (100 km away) for abdominal ultrasound and full laboratory workup, or straight away to the only tertiary hospital with capabilities for right heart catheterization (RHC) in Mozambique’s capital (1000 km away). The average time for all these procedures is around six months to one year [Figure 1].

## 3. Unique Patterns of Pulmonary Hypertension in African Children

Our patient’s trajectory unveils the long pathway for PH diagnosis in children living in Sub-Saharan Africa, where late presentation reflects the poor socio-economic context, low health literacy, poor awareness of health providers, and the unpreparedness of the health services to diagnose this condition and its risk factors [10]. Indeed, there is a unique profile of causes and risk factors for PH in Sub-Saharan Africa. Untreated congenital heart disease, valvular rheumatic heart disease, and cardiomyopathy are common causes in children [11,12,13]. The delayed diagnoses of preventable causes and the long-term synergistic effects of infectious comorbidities—such as HIV, schistosomiasis, and tuberculosis (TB) [10,11]—seem to be key in determining high PH morbidity and mortality in LMIC, but have not been systematically assessed.

## 4. Epidemiology of Infectious Pulmonary Vascular Disease in Sub-Saharan Africa

HIV, TB, and schistosomiasis should be considered in any case of PH. Importantly, PAH associated with schistosomiasis (PAH-Sch) and with HIV (PAH-HIV) cannot be distinguished from idiopathic PAH based on clinical, echocardiographic, or hemodynamic features. In Mozambique, there are structured vertical programs supported by global funding mechanisms that allow decentralization of diagnosis and management of patients with HIV and TB in the public health sector. This is carried out through a model where clinics led by trained medical officers and nurses screen patients, perform laboratory or rapid tests, and prescribe drugs using clinical algorithms. All procedures are at no cost to the patients. In contrast, for schistosomiasis, the pathway to diagnosis and access to interventions is not established, partially due to a lack of standardized algorithms, related to uncertainty regarding the exact pathogenic mechanisms of pulmonary arterial hypertension (PAH) associated with this parasite [14].

The prevalence of PH among people with HIV in Sub-Saharan Africa seems to be higher when compared to other regions, varying between 5% and 14% [15,16]. The prospective and multinational Pan African Pulmonary Hypertension Cohort (PAPUCO) recruited 220 consecutive patients (209 adults and 11 children; 97% of African descent) from nine specialist centers in four countries, providing insights into the profile of PH and the determinants of unfavorable survival outcomes in the region, some readily evaluable and amenable to modification [17,18]. Patients with HIV newly diagnosed with PH were younger and, most commonly, had a previous diagnosis of tuberculosis and a worse survival rate [11]. HIV infection was associated with decreased survival at 6 months [18]. Considering the fact that HIV-related PAH shares histological characteristics with other types of PAH and is ameliorated by antiretroviral therapy, there is a need to understand the role of associated infections in determining poorer outcomes of antiretroviral therapy in African cohorts [15].

PH may occur in active tuberculosis and as part of post-tuberculosis lung disease (PTLD) as shown in South Africa. Out of 100 non-healthcare-seeking adults who had successfully completed TB treatment (71 males; mean age: 42 years), 9 (9%) had probable PH, and a combined PH prevalence of 4% was found among 100 patients (mean age: 37.1 years, 58% male; 46% HIV positive) with a first documented episode of TB, who were in the second half of treatment or had recently completed it [19]. A systematic review and meta-analysis of 14 post-TB studies (out of the 23 studies included) assessed by right heart catheterization or echocardiography revealed a prevalence of PH at 67.0% (95% CI 50.8–81.4) in patients with chronic respiratory failure, 42.4% (95% CI 31.3–54.0) in hospitalized or symptomatic patients, and 6.3% (95% CI 2.3–11.8) in non-healthcare-seeking outpatients (I^2^ = 96%); patients with active TB had a lower estimated prevalence (9.4%, 95% CI 6.3–13.0), I^2^ = 84%) [20].

## 5. Challenges for the Diagnosis of *Schistosoma*-Associated Pulmonary Arterial Hypertension

In regions at high risk of exposure to schistosomiasis, delayed presentation increases the challenges to accurately diagnosing PAH-Sch, which may occur in about 6.1% of those chronically infected, particularly with the species *Schistosoma mansoni*. In addition to mechanical obstruction of the pulmonary vasculature by parasite eggs, pre-existing hepatosplenic disease due to *Schistosoma mansoni* infection seems to be a requirement, causing porto-pulmonary hypertension and/or allowing egg embolization to the lung via portocaval shunts [14]. The diagnosis of PAH-Sch may require serial blood tests, multimodal imaging (for monitoring of heart and pulmonary artery dilatation), and rectal biopsies—the availability of such procedures is restricted to tertiary hospitals [14]. Similarly, access to targeted pharmacological therapy, such as phosphodiesterase type 5 inhibitors and endothelin receptor antagonists, may be limited to urban centers.

In our patient, tuberculosis and HIV had been ruled out, and the diagnosis of schistosomiasis had been considered but was difficult to confirm in the rural setting. This remains the biggest challenge to diagnosing PAH-Sch, which includes ensuring evidence of previous schist-PAH [21] and demonstrating the presence of prehepatic portal hypertension [22,23,24,25,26]. Serological tests for schistosomiasis can be helpful [27], but two or more assays need to be performed in parallel due to limitations in test sensitivity [28]. Specific tests are highly sensitive and cost-effective methods for assessment of *Schistosoma*-induced fibrosis [29], but people living in endemic areas are likely to have positive serological tests due to prior infections. In addition, the absence of eggs in the stool does not rule out PAH-Sch [30].

In the absence of specific clinical and biochemical morbidity markers and an expert liver ultrasound in the rural setting, we used indirect methods—such as evidence of peripheral blood eosinophilia, hepatomegaly, and splenomegaly—as clinical and biochemical markers of schistosomiasis and liver fibrosis. Of note, hyperosinophilia is commonly found in African children linked to other parasites. A key aspect in confirming our diagnostic hypothesis is the presence of periportal fibrosis at abdominal ultrasonography, irrespective of previous treatment for schistosomiasis or positive screening for *Schistosoma* eggs by routine methods [31,32]. Mozambique school health program includes periodic mass administration of praziquantel in public schools. While the patient could be scheduled for an abdominal ultrasound and stool examination in the closest referral hospital (100 km), this health facility does not perform rectal biopsy or RHC and would not have the necessary skilled personnel to institute specific drug therapy, and therefore, a transfer to the capital city is being considered. Referral procedures need to be organized by the medical team and include obtaining parents’ informed consent for RHC, guiding the family in obtaining the national identification card to be eligible for social security support for the trip, securing a family member to travel with the child, and contacting the recipient health facility to plan the diagnostic procedures. These procedures take several months, and some children are lost in the process—mostly because the parents do not accept or are not able to leave the other children alone at home.

## 6. The Need for Innovation and Holistic Approaches to Schistosomiasis Control

Over 97% of PH patients live in endemic areas for schistosomiasis. The epidemiological importance of PAH-Sch contrasts with the lack of specific guidance to address these cases in PH guidelines globally and in endemic areas. Simplified guidelines and tailored task-shifting strategies have been developed for early screening and diagnosis of left heart disease PH in rural Africa [33]. Screening for infectious PH has also been incorporated in pragmatic algorithms for low-income settings [34], in an attempt to foster awareness of health providers, establishment of referral pathways for efficient flow of patients between different levels of the health system, and provision of the needed diagnostics and medicines to allow decentralization of PH diagnosis in Sub-Saharan Africa. However, the complex patterns of multimorbidity and the unpreparedness of health facilities make it difficult to implement such algorithms in most rural areas in Sub-Saharan Africa. Moreover, in addition to social determinants of health playing a major role in determining the profile of iPVD, together with health system factors described above, they contribute to the underrepresentation of patients from LMIC in clinical trials and registries for PAH.

Overall, this case illustrates the challenges faced in remote areas of low- and middle-income countries to diagnose infectious PVD in rural Mozambique. It highlights how much social determinants of health and health services’ configuration influence the capacity to diagnose and treat PH in LMIC. However, it should be stressed that these diseases are preventable, and that continuous efforts to address their high prevalence in low-income settings must continue. Public health measures, environmental interventions, and changes in individual behavior are needed to reduce the transmission of HIV, tuberculosis, and schistosomiasis. While there have been campaigns of mass administration of praziquantel to schoolchildren in afflicted communities [35], the impact of these interventions has not been systematically reported. In African countries where these evaluations have been made, hotspots of infections persist [36,37,38], providing evidence for targeted sub-district-level treatment and suggesting the need for integrated control interventions incorporating behavioral change, preventive chemotherapy, and vector control. Aribodor et al. noted that in Nigeria [39], infection levels after mass administration of anti-parasitic drugs varied by location—with socio-economic status and inadequate water, sanitation, and hygiene infrastructure contributing to transmission risk—income, and practice of open defecation.

To improve early diagnosis of PAH-Sch, social and health systems’ determinants need to be addressed, including (i) poor health literacy and risk behavior of the communities; (ii) limited access to diagnostic tools for confirmation of schistosomiasis outside major referral urban hospitals; (iii) low awareness of health providers; (iv) limited access to right heart catheterization and pathology laboratories for deep phenotype of PH; and (v) low access to target therapies.

Key priorities for endemic areas such as Mozambique are to pilot interruption of transmission in hotspots; enhance integrated water, sanitation, and hygiene (WASH) measures; implement focal snail control; and pilot pediatric praziquantel for treatment of schistosomiasis in preschool-aged children. The advent of the point-of-care circulating cathodic antigen (POCCCA) test, in addition to Kato–Katz, has been improving the mapping accuracy and detection of low-intensity infections in Rwanda [39], allowing identification of hotspots, expansion of mass drug administration coverage, adoption of One Health approaches to address socio-environmental drivers, and implementation of WASH interventions, all enablers for targeted interventions to eliminate schistosomiasis.

## 7. Conclusions

There are major gaps in knowledge regarding the best epidemiological, clinical, and laboratory methods for the detection of iPVD. Access to specific testing, RHC, and target interventions is limited to specialized centers in Sub-Saharan Africa, hampering early diagnosis of infectious PH. Hence, there is a need for pragmatic guidelines for screening based on echocardiography performed by non-specialists, supported by simplified algorithms and point-of-care testing of vulnerable populations. Ultimately, improvements in geomapping of high transmission areas, accurate diagnosis, and tailored social and behavior change communication should improve the capacity to advocate for early-stage PAH specific therapy and drug developments to reduce the burden of iPVD in Africa.

## Data Availability

The raw data supporting the conclusions of this article will be made available by the authors on request.
